# Complete linear optical isolation at the microscale with ultralow loss

**DOI:** 10.1038/s41598-017-01494-w

**Published:** 2017-05-08

**Authors:** JunHwan Kim, Seunghwi Kim, Gaurav Bahl

**Affiliations:** Mechanical Science and Engineering, University of Illinois at Urbana-Champaign, Urbana, Illinois USA

## Abstract

Low-loss optical isolators and circulators are critical nonreciprocal components for signal routing and protection, but their chip-scale integration is not yet practical using standard photonics foundry processes. The significant challenges that confront integration of magneto-optic nonreciprocal systems on chip have made imperative the exploration of magnet free alternatives. However, none of these approaches have yet demonstrated linear optical isolation with ideal characteristics over a microscale footprint – simultaneously incorporating large contrast with ultralow forward loss – having fundamental compatibility with photonic integration in standard waveguide materials. Here we demonstrate that complete linear optical isolation can be obtained within any dielectric waveguide using only a whispering-gallery microresonator pumped by a single-frequency laser. The isolation originates from a nonreciprocal induced transparency based on a coherent light-sound interaction, with the coupling originating from the traveling-wave Brillouin scattering interaction, that breaks time-reversal symmetry within the waveguide-resonator system. Our result demonstrates that material-agnostic and wavelength-agnostic optical isolation is far more accessible for chip-scale photonics than previously thought.

## Introduction

Ideal optical isolators should exhibit complete linear isolation – where completeness implies perfect transmission one way (i.e. zero forward insertion loss) and zero transmission in the opposite direction – without any mode shifts, frequency shifts, or dependence on input signal power. In practice, isolators should also exhibit a broadband isolation response for robustness and usability across a wide range of applications. To date, the best method for achieving optical isolation with these characteristics has been through Faraday rotation via the magneto-optic response in gyrotropic materials^1, 2^. Unfortunately, this well-established technique^3^ has proven challenging to implement in chip-scale photonics due to fabrication complexity, difficulty in locally confining magnetic fields, and significant material losses^4–7^.

In light of this challenge, several non-magnetic alternatives for breaking reciprocity^3^ have been explored both theoretically^8–13^ and experimentally^14–19^. State-of-the-art experimental implementations of these alternatives have succeeded in meeting various metrics of contrast, linearity, and bandwidth, but complete isolation with ultralow forward loss has remained elusive. Nonlinearity-based isolators can be broadband but are fundamentally dependent on input field strength^16, 18^ and hence do not produce a linear isolation response^20^. Dynamic modulation^8, 10^, is a powerful approach that can generate linear isolation over potentially wide bandwidth, but current microscale demonstrations are still constrained by very large forward insertion loss and low contrast^15, 17^. These limitations could be overcome in macro-scale implementations^21^. Finally, the use of Brillouin acousto-optic scattering to induce unidirectional optical loss^9, 22^ is very promising, as it can generate a linear wide-band isolation response. In practice, this technique requires a large product of scattering gain and waveguide length^14^, which could soon be realized through advancements in on-chip Brillouin gain^23–25^. To date, however, there has been no SBS isolator demonstrated having a sub-millimeter footprint. A comparison of state-of-the-art experimental results on non-magnetic microscale isolation can be found in Table S1 of the Supplement. In this paper we emphasize forward loss, as it is an especially strong motivator for chip-scale photonics where we consistently strive to lower the product of size, weight, and power (SWaP) parameters.

Recently, a fundamentally different path to obtain nonreciprocal optical transport has emerged, by exploiting opto-mechanically induced transparency^26–28^. These nonreciprocal effects are based on destructive optical interference via a non-radiative acoustic coherence within a resonator-waveguide system, and are acousto-optic analogues of electromagnetically induced transparency (EIT). To date, however, only subtle nonreciprocity has been demonstrated by these optomechanical methods, without any expression or demonstration of a path to achieving complete isolation with ultra-low forward loss, both of which are requirements for practical use. We focus our study on the nonreciprocal Brillouin scattering induced transparency (BSIT) mechanism^26^, in which momentum conservation requirement between photons and phonons helps break time-reversal symmetry for light propagation. More importantly, BSIT uniquely permits two major technical results that we demonstrate in this work. We show theoretically that when operating within the strong acousto-optical coupling regime (aided by the resonant pump) the BSIT system enables theoretically lossless transmission of light in the forward direction in a waveguide, while maintaining complete absorption in the reverse direction – the condition of complete linear isolation. Second, the non-zero momentum of the traveling phonons involved in BSIT permits independent forward/reverse reconfiguration of the isolation effect, in contrast to the zero-momentum mechanical excitations in optomechanically induced transparency^28^ that can couple forward and reverse propagating light. Experimentally, we demonstrate a device operating very close to the strong coupling regime and capable of generating a record-breaking 78.6 dB of isolation contrast per 1 dB of forward insertion loss within the induced transparency bandwidth. Since the underlying interaction is available in all dielectrics, this isolation effect can in principle be implemented using any waveguide and resonator materials available in photonics foundries.

## Achieving Complete Linear Isolation

### Qualitative description

**Figure 1:**
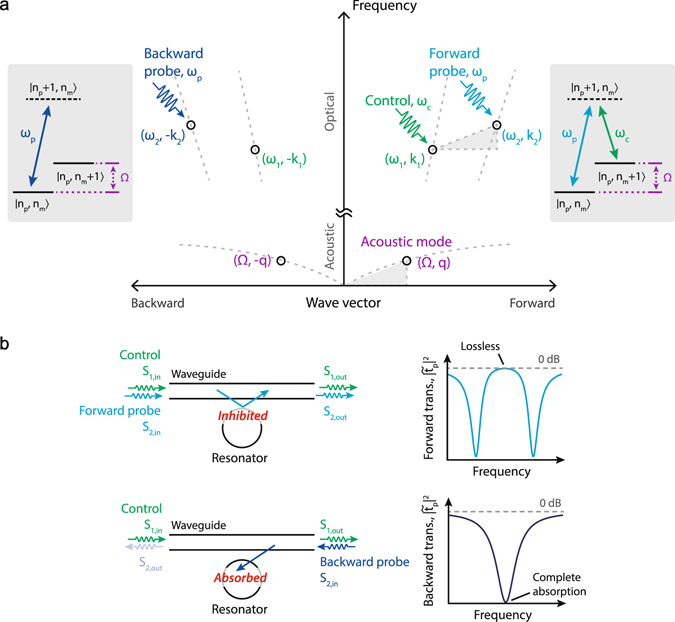
Achieving optical isolation through non-reciprocal Brillouin scattering induced transparency in a whispering-gallery resonator: (**a**) The interference of excitation pathways in the BSIT system are described through an energy-level picture (grey boxes), using probe photon number *n*_*p*_ and phonon number *n*_*m*_. Absorption of a probe photon into the (*ω*_2_, k_2_) optical resonance is modeled as an effective transition $|{n}_{p},{n}_{m}\rangle \to |{n}_{p}+1,{n}_{m}\rangle$. In presence of the control field, the probe photon could scatter to the lower resonance (*ω*_1_, k_1_) while adding a mechanical excitation in (Ω, q), which is an effective transition to state $|{n}_{p},{n}_{m}+1\rangle$. However, the coherent anti-Stokes scattering of the control field from this mechanical excitation would generate an interfering excitation pathway for the original state $|{n}_{p}+1,{n}_{m}\rangle$. This process is analogous to EIT and results in a window of transparency for the forward optical probe, inhibiting the original $|{n}_{p},{n}_{m}\rangle \to |{n}_{p}+1,{n}_{m}\rangle$ absorption transition. The necessary momentum matching requirement, not visible in the energy diagram, is represented using the dispersion relation (middle) to elucidate the breaking of time-reversal symmetry for the probe signal. (**b**) We implement this mechanism using a waveguide and a whispering gallery resonator, in which probe signals tuned to either of the (*ω*_2_, ±k_2_) optical resonances are typically absorbed by the resonator under the critical coupling condition. The presence of a forward control field, however, creates the BSIT interference^26^, only for forward probe signals and inhibits absorption. Under strong acousto-optical coupling, the waveguide-resonator system is rendered lossless at the original resonance.

Let us first qualitatively discuss how ideal optical isolation can be achieved by means of the BSIT light-sound interaction in dielectric resonators^26, 27^. We consider a whispering-gallery resonator having two optical modes (*ω*_1_, k_1_) and (*ω*_2_, k_2_) that are separated in (*ω*, k) space by the parameters of a high coherence traveling acoustic mode (Ω, q). This is the requisite phase matching relation for BSIT (Fig. 1a), indicating that phonons enable coupling of the photon modes through photoelastic scattering. We stress here that the two modes should belong to different mode families of the resonator in order to ensure that scattering to other optical modes from the same phonon population is suppressed. When this system is pumped with a strong ‘control’ field on the lower optical resonance (*ω*_1_, k_1_), an EIT-like optomechanically induced transparency^29, 30^ appears within the higher optical resonance (*ω*_2_, k_2_), due to coherent interference originating from the acousto-optical interaction^26, 27^.

A description of this interference can be presented both classically^26^, or through by a quantum mechanical approach^27^. Briefly, one can consider signal or ‘probe’ photons arriving from the waveguide at frequency *ω*_2_ that are on-resonance and being absorbed by the resonator mode (*ω*_2_, k_2_). When the control field is present in a BSIT phase-matching situation, these probe photons could scatter to (*ω*_1_, k_1_) causing a mechanical excitation of the system. However, anti-Stokes scattering of the strong control field from this mechanical excitation will generate a phase-coherent optical field that interferes destructively with the original excitation of the mode at (*ω*_2_, k_2_). The result is a pathway interference that is measured as an induced optical transparency in the waveguide, where no optical or mechanical excitation takes place, and the resonant optical absorption is inhibited (Fig. 1b - top). The strength of this interference is set by the intensity of the control laser. The phase of the mechanically dark mode is instantaneously set by the phases of the control and probe optical fields, and does not require phase coherence between them. It is crucial, however, to note that this transparency in BSIT only appears for probe signals co-propagating with the control laser. Probe light in the counter propagating i.e. time-reversed direction, on the other hand, occupies the high frequency optical mode with parameters (*ω*_2_, −k_2_). For BSIT to occur in this case, an acoustic mode having parameters (Ω, −(k_1_ + k_2_)) would be required for compensating the momentum mismatch between the forward control and backward probe optical modes. However, since such an acoustic mode is not available in the system, no interaction occurs for the counter-propagating probe and the signal is simply absorbed into the resonator (Fig. 1b - bottom).

### Classical treatment of the system

The classical field equations for coupled light and sound in this waveguide-resonator system are presented in the Supplement. The transmission coefficient ${\tilde{t}}_{p}$ of the probe laser field can be derived as:

1 $${\tilde{t}}_{p}=\frac{{s}_{\mathrm{2,}{\rm{out}}}}{{s}_{\mathrm{2,}{\rm{in}}}}=1-\frac{{\kappa }_{{\rm{ex}}}}{({\kappa }_{2}\mathrm{/2}+j{{\rm{\Delta }}}_{2})+{G}^{2}/({{\rm{\Gamma }}}_{B}\mathrm{/2}+j{{\rm{\Delta }}}_{B})}$$

where ${s}_{i,{\rm{in}}}$ and ${s}_{i,{\rm{out}}}$ are the optical driving and output fields in the waveguide (Fig. 1b) at the control (*i* = 1) and probe (*i* = 2) frequencies. *G* is the pump-enhanced Brillouin coupling rate manipulated by the control optical field ${s}_{\mathrm{1,}{\rm{in}}}$ in the waveguide via the relation $G=|{s}_{\mathrm{1,}{\rm{in}}}\,\beta \,\sqrt{{\kappa }_{{\rm{ex}}}}/({\kappa }_{1}\mathrm{/2}+j{{\rm{\Delta }}}_{1})|$. Here *β* is the acousto-optic coupling rate, *κ*_*i*_ are the loaded optical loss rates, Γ_*B*_ is the phonon loss rate, and ${\kappa }_{{\rm{ex}}}$ is the coupling rate between the waveguide and resonator. The loaded optical loss rates are defined as ${\kappa }_{i}={\kappa }_{i,{\rm{o}}}+{\kappa }_{ex}$ where ${\kappa }_{i,{\rm{o}}}$ is the loss rate intrinsic to the optical mode. The ${{\rm{\Delta }}}_{i}$ parameters are the field detunings, with subscript *B* indicating the acoustic field. This response matches the system of optomechanically induced transparency (OMIT)^29, 30^, with the exception that the pump field is also resonant and the coupling rate *β* is dependent on momentum matching. As we explain later, the pump resonance in BSIT significantly enhances the maximum coupling rate *G* achievable in contrast to single-mode OMIT systems.

Equation (1) is key to understanding how an ideal optical isolator can be obtained. First, we examine the case of no acousto-optic coupling *G* = 0, resulting from either modal mismatch (*β* = 0) or zero applied control laser power (${s}_{\mathrm{1,}{\rm{in}}}=0$). In this case Eq. (1) exhibits a well-known Lorentzian shaped transmission dip implying that the probe optical field in the waveguide is simply absorbed by the resonator^31^. Critical coupling between resonator and waveguide is enabled when ${\kappa }_{{\rm{ex}}}={\kappa }_{\mathrm{2,}{\rm{o}}}$ and results in complete absorption of the probe light from the waveguide at resonance (${{\rm{\Delta }}}_{2}=0$). With critical coupling in place, let us now introduce the effects of the acousto-optic coupling. For very large acousto-optic interaction strength, i.e. $G\to \infty$, Eq. (1) indicates that we recover perfect transmission ${|{\tilde{t}}_{p}|}^{2}=1$ even when the waveguide and resonator are critically coupled. Forward propagating probe light in the waveguide, co-propagating with the control laser, can thus transmit perfectly with no absorption at resonance in the ideal case. At the same time, we have no Brillouin coupling ($\beta =0$) for counter-propagating control and probe optical fields due to the momentum mismatch as indicated previously. This implies that, for a counter-propagating probe, the system remains in the critical coupling region resulting in complete absorption. Since forward probe signals transmit with zero absorption, and backward probe signals are completely absorbed (Fig. 1b), this system is an ideal linear isolator at the transparency resonance.

**Figure 2:**
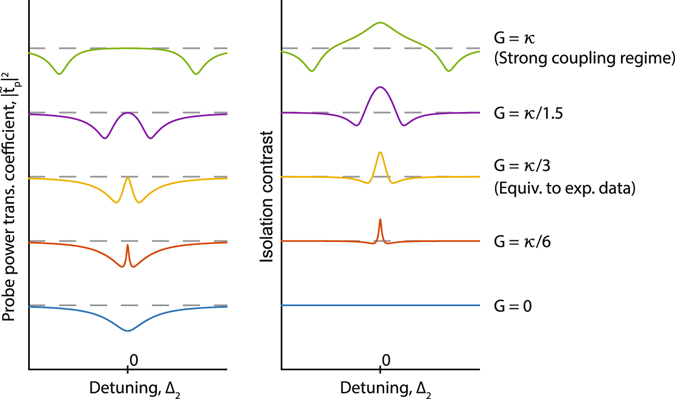
Evolution of the transparency and isolation contrast as a function of pump-enhanced Brillouin coupling *G*. In the weak coupling regime ($G\ll \kappa$), the transparency linewidth and contrast bandwidth are defined by the acoustic linewidth Γ_*B*_^26^. As coupling *G* increases, the isolation contrast improves, bandwidth is expanded and the optical mode with transparency appears as a splitted mode. In the strong coupling regime, the isolation bandwidth is independent of the acoustic mode and is instead defined by optical mode linewidth *κ* only. The dashed lines indicate the perfect transmission baseline (left) and zero isolation contrast (right) respectively.

A more practically accessible case is $G\ge {\kappa }_{2}$, also known as the strong coupling regime^32^, where the induced transparency grows to the width of the optical mode. Strong coupling can be reached for high coherence phonon modes (small Γ_*B*_) with large acousto-optic coupling *β* and large control driving field ${s}_{\mathrm{1,}{\rm{in}}}$. The evolution of the optical transparency and isolation contrast with increasing coupling *G* is illustrated in Fig. 2. In the weak coupling regime ($G\ll {\kappa }_{2}$) the isolation contrast is defined roughly by the linewidth of the phonon mode. As *G* increases the transparency window broadens until eventually reaching the strong coupling regime where the isolation contrast bandwidth reaches a maximum equaling the optical loss rate *κ*_2_, as long as the acoustic frequency is higher than this value. Thus, the isolation bandwidth can be improved to the several GHz range if a higher frequency acoustic mode is used^33^, in conjunction with a low-Q (high *κ*_2_) optical mode, and the reduction in coupling is compensated by other means^34^. In this regime, we also achieve the desired ultra-low forward insertion loss. Such large transparency can also be interpreted as the splitting of the optical mode^35^. The absence or minimization of forward loss necessarily implies linear optical response at frequency *ω*_2_ without any nonlinearity or mode conversion.

## Results and Discussion

### Demonstration of high-contrast ultralow-loss isolation

**Figure 3:**
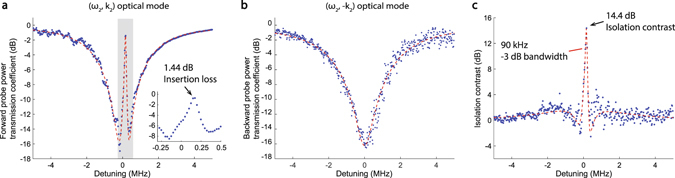
Experimental observation of extremely low insertion loss linear optical isolation. (**a**) Probe power transmission coefficient ${|{\tilde{t}}_{p}|}^{2}$ is measured in the forward direction through the waveguide near the (*ω*_2_, k_2_) mode, with fixed 66 *μW* pump power dropped into the (*ω*_1_, k_1_) mode. The forward probe power transmission coefficient through the waveguide shows only 1.44 dB insertion loss within the transparency. The phonon mode frequency is 145 MHz. (**b**) The (*ω*_2_, −k_2_) optical mode measured by the backward probe does not exhibit the induced transparency, resulting in conventional absorption of the probe signal by the resonator. (**c**) The optical isolation contrast is evaluated as the difference between forward and backward power transmission coefficients. Here we calculate 14.4 dB peak contrast with a −3 dB bandwidth of 90 kHz. Isolation exists over 470 kHz.

We experimentally demonstrate ultra-low loss optical isolation (Fig. 3) in the waveguide-resonator system by probing optical transmission through the waveguide in the forward and backward directions simultaneously (see Methods). A resonator of diameter 170 μm is used to guarantee the natural existence of multiple triplets of acoustic and optical modes that satisfy the phase-matching condition for BSIT.

The requisite BSIT phase-matching is first experimentally verified by strongly driving the (*ω*_2_, k_2_) optical mode and observing spontaneous and stimulated Stokes Brillouin scattering into the lower mode (*ω*_1_, k_1_) in the forward direction^33^. Subsequently, we drive the (*ω*_1_, k_1_) optical mode with a strong control laser (<1 mW) and use a weak co-propagating probe laser to measure the power transmission spectrum across the high frequency optical mode (*ω*_2_, k_2_) revealing the induced transparency window. The control laser detuning and power are adjusted in order to maximize the power transmission within the transparency peak. Experimental measurements of the probe power transmission ${|{\tilde{t}}_{p}|}^{2}$ in both forward and backward directions are presented in Fig. 3. To show optical isolation, the same measurement is taken in the forward and backward directions while the constant control driving field ${s}_{\mathrm{1,}{\rm{in}}}$ is supplied in the forward direction only. In this experiment the two selected optical modes of the resonator have linewidth ${\kappa }_{1}\approx {\kappa }_{2}\approx 4.1$ MHz, and are spaced approximately 145 MHz apart. They are coupled by means of a 145 MHz acoustic mode of intrinsic linewidth Γ_B_ ≈ 12 kHz. Through finite element simulations, we estimate that the acoustic mode corresponds to a first order Rayleigh surface acoustic exictation having an azimuthal order of M = 24. At a diameter of 170 *μm*, this translates to an acoustic momentum of *q* = 0.28 μ*m* ^−1^ and ensures breaking of interaction symmetry for co-propagating and counter-propagating probe fields (Fig. 1a).

As seen in Fig. 3a the system exhibits very low forward insertion loss (1.44 dB) at the peak of induced transparency region for 66 *μW* control laser power absorbed to the resonator (power launched in fiber is 680 *μW*). This corresponds to an experimentally calculated pump-enhanced Brillouin coupling of $G\approx {\kappa }_{2}/12$. At this point, the acoustic mode has an effective linewidth of 80.4 kHz due to Brillouin cooling^36^. Simultaneous measurement of backward probe power transmission (Fig. 3b) shows only the absorption spectrum of the unperturbed (*ω*_2_, −k_2_) optical mode, generating a power transmission loss of 15.8 dB in the waveguide. Subtraction of the forward and backward measurements provides a measure of the optical isolation contrast, which is 14.4 dB here with ~90 kHz full width at half maximum (Fig. 3c).

Since the forward insertion loss is very low (zero in the ideal theoretical case), the isolation contrast is primarily determined by the proximity of the waveguide-resonator coupling to the critical coupling condition, which if achieved would yield infinite isolation contrast. Achieving critical coupling ${\kappa }_{{\rm{ex}}}={\kappa }_{\mathrm{2,}{\rm{o}}}$ in non-integrated waveguide-microsphere systems is very challenging due to multimode waveguiding in the taper, thermal drifts during the experiment, and vibrational or mechanical stability issues. Previously, up to 26 dB of signal extinction has been experimentally demonstrated in a fiber taper-microsphere system^37^. In the future, ideal isolation may be approached if the waveguide and resonator are integrated on-chip, since most mechanical issues can be eliminated and the interacting modes can be designed precisely. Alternatively, applications that require high contrast may employ multiple isolators in series with minimal penalty due to the extremely low insertion loss in this system. It is thus appropriate to compare performance of different isolators by referencing the achieved contrast to 1 dB forward loss. The data shown in Fig. 3 indicates this figure of merit of approximately 10 ${{\rm{dB}}}_{{\rm{isolation}}}/{{\rm{dB}}}_{{\rm{loss}}}$ (units preserved for clarity, indicating 14.4 dB constrast vs 1.44 dB forward loss).

**Figure 4:**
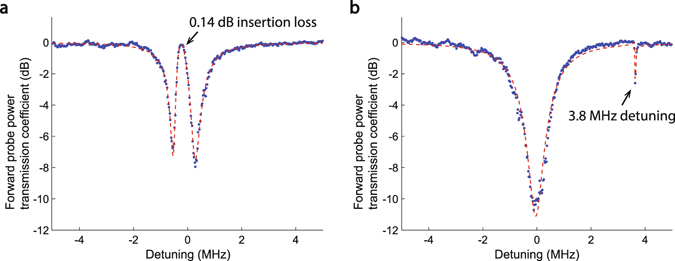
Demonstration of ultra-low forward insertion loss with stronger coupling *G*. (**a**) Here, we use a triplet of optical and acoustic modes with an optical mode separation or acoustic frequency of 164.8 MHz. Pump-enhanced Brillouin coupling rate *G* is much higher due to better acousto-optic modal overlap and 235 *μW* power absorbed into the control mode. This results in $G\approx \kappa \mathrm{/3}$ causing the forward insertion loss within the transparency to decrease to only 0.14 dB. The isolation bandwidth also increases to approximately 400 kHz. (**b**) The transparency-free (*ω*_2_, k_2_) optical mode is observable by detuning the control laser from the (*ω*_1_, k_1_) optical mode, which also detunes the scattered light.

Theory indicates that much lower forward insertion loss can be obtained if much higher coupling rate *G* is arranged, either by lowering the loss rates of the optical modes, or by using higher control laser power. Fortunately, a special feature of two-mode systems such as BSIT^38^ is the resonant enhancement of the intracavity pump photons in mode *ω*_1_, which enables much easier access to the strong coupling regime. Nonreciprocity based on single-mode OMIT^28^ does not possess this feature and it is thus impractical to expand the isolation bandwidth and reach the ultra-low loss regime. Making use of this resonant enhancement, in Fig. 4a we show a system nearly reaching the strong coupling regime with $G\approx \kappa /3$, exhibiting only 0.14 dB forward insertion loss (96.8% transmission) and isolation contrast estimated at 11 dB. Here, 235 *μW* control power is coupled to the resonator (700 *μW* launched in fiber). The unmodified optical mode absorption can be easily observed by detuning the control laser such that the interference is generated outside the optical mode (Fig. 4b). This result indicates that the strong coupling regime is also within the reach of this silica waveguide-resonator system^38^. The isolation figure of merit (referenced to 1 dB insertion loss) for the Fig. 4 result is quantified at 78.6 ${{\rm{dB}}}_{{\rm{isolation}}}/{{\rm{dB}}}_{{\rm{loss}}}$. This compares extremely well to commercial fiber-optic Faraday isolators whose figures of merit typically range between 60–100 ${{\rm{dB}}}_{{\rm{isolation}}}/{{\rm{dB}}}_{{\rm{loss}}}$, and far exceeds the capabilities demonstrated till date by any other non-magnetic microscale optical isolation approach. As shown in Supplementary Table S1, our achieved contrast exceeds the next best microscale experimental result in non-magnetic optical isolation^19^ by nearly 7 orders-of-magnitude (69.5 dB difference, i.e. 78.6 dB vs 9.09 dB) per 1 dB of insertion loss.

### Independent reconfiguration of optical isolation

**Figure 5:**
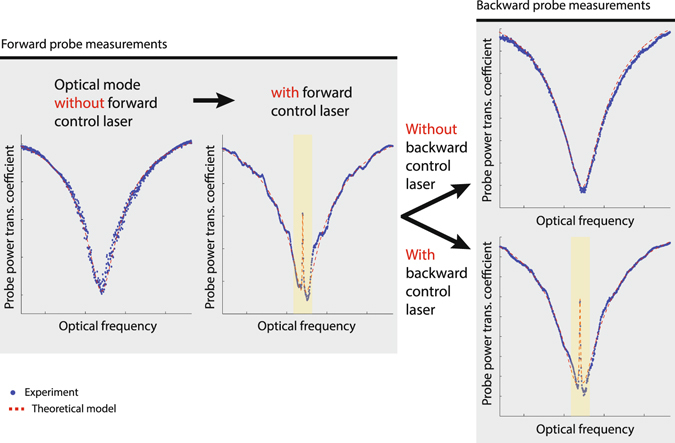
Demonstrating reconfigurable optical isolation. Increasing the control laser power in the forward direction, we observe the appearance of the acousto-optical transparency. While transparency is enabled in the forward direction, we can switch on and off the transparency in the backward direction using a separate backward propagating control laser. The red dashed line represents a fit using theoretical model for induced transparency.

Finally, we also demonstrate the optical reconfigurability of the isolation direction by means of independent control lasers that propagate in opposite directions. This is demonstrated through an experiment (Fig. 5) where the the control laser field is sequentially provided in the forward direction only, backward direction only, and in both directions simultaneously. Since the forward and backward directions in a whispering-gallery resonator are nominally decoupled and the phonon mode also has an associated directionality (i.e. momentum), the transparency is independently observed in the directions in which a control laser field is supplied.

Figure 5 shows that when no control field is provided, the anti-Stokes optical mode is a simple Lorentzian shaped dip. However, when the control laser is supplied in the forward direction, a transparency is observed by the forward probe. While this transparency is sustained in the forward direction, we can independently switch on and off the transparency in the backward direction. This is demonstrated by probing the anti-Stokes optical mode in the backward direction with and without a backward control laser, which results in an optical mode with and without transparency respectively. Such reconfigurable transparency has never previously been demonstrated in any other optical or opto-mechanical system.

OMIT-based nonreciprocity^28^ does not possess the capability of fully independent reconfiguration since both forward and reverse optical signals interact with the same zero-momentum vibrational mode. Thus photon conversion can occur through an optomechanical dark mode^39^ shared between forward and reverse pumps, i.e. forward (reverse) sources can modify light propagation in the reverse (forward) direction.

## Conclusions

Achieving complete linear optical isolation through opto-mechanical interactions that occur in all media, irrespective of crystallinity or amorphicity, material band structure, magnetic bias, or presence of gain, ensures that the technique could be implemented in nearly any photonic foundry process with any optical material. Example systems that could support this isolation approach are released optomechanical resonators with co-integrated waveguides such as those shown in ref. 40. Since the isolation bandwidth demonstrated here is relatively narrow (about 400 kHz), but is wavelength agnostic, this approach must be tailored for particular photonic device applications. However, we must emphasize that the maximum bandwidth of this isolation approach under strong acousto-optical coupling is only limited by the optical mode linewidth *κ*_2_, allowing future improvement in isolation bandwidth to several GHz with the use of low optical Q-factor modes and higher acoustic frequencies. In contrast to all previous works, this induced transparency approach ensures that bidirectional signals are attenuated by default, and only unidirectional transport is enabled when the control optical stimulus is applied. This scheme additionally ensures protection for the system to be isolated in case of failure of the control source, and allows the possibility of dynamic optical shuttering. The absence of magnetic or radiofrequency electromagnetic driving fields make this approach particularly useful for chip-scale cold atom microsystems technologies, for both isolation and shuttering of optical signals, and laser protection without loss.

## Methods

### Waveguide-Resonator System

We experimentally demonstrate ultra-low loss optical isolation by probing light transmission through the waveguide in the forward and backward directions simultaneously. In our experiment, a tapered optical fiber waveguide is fabricated by linear tension drawing of SMF-28 fiber while being heated with a hydrogen flame^41^, till the point that the tapered waveguide diameter is comparable to the laser wavelength and supports only a single optical mode with significant evanescent field. With adiabatic tapering^42^ the loss associated with this waveguide can be made as low as 0.003 dB^43^. We employed a resonator of diameter 170 μm to guarantee the natural existence of multiple triplets of acoustic and optical modes that satisfy the phase-matching condition for BSIT, although smaller resonators may also be used. The microsphere resonator is fabricated by reflow of a single-ended optical fiber taper using an arc discharge. The fiber mode is coupled to the resonator by means of evanescent field overlap with the resonator’s whispering gallery modes. The optical coupling rate is controlled using distance with a piezo-nanopositioner.

### Experimental setup

**Figure 6:**
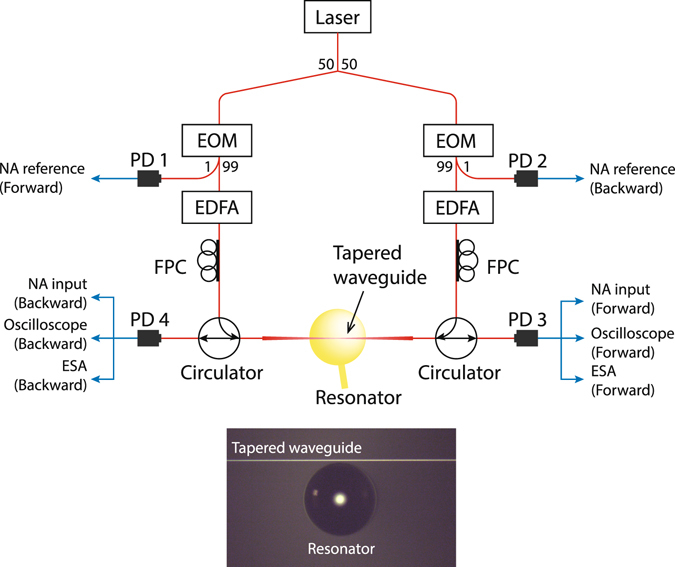
Experimental setup for simultaneous forward and backward probe transmission measurements is shown. We use a matched set of optical components including the electro-optic modulator (EOM), the erbium-doped fiber amplifier (EDFA), the fiber polarization contrller (FPC), the circulator and the photodetectors (PD) for the forward and backward measurements. Light is coupled to the resonator via tapered waveguide. An electronic network analyzer (NA) performs ratiometric measurements of NA reference and NA input signals (marked in figure) in the forward and backward directions. The electrical spectrum analyzer (ESA) is also used to observe the acoustic phonon mode by measuring the beat note generated by the control laser and Brillouin light scattering by the phonons.

The experimental setup used for the simultaneous forward and backward measurements is shown in Fig. 6. We employ a 1520–1570 nm tunable external cavity diode laser (ECDL) to generate the control and probe laser fields. This laser source is first split into the forward and backward directions using a 50:50 splitter. Electro-optic modulators (EOM) are employed as variable optical attenuators in dc mode (i.e. by adjusting the bias voltage) for manipulating control laser power in either direction. The probe laser is also derived from the control laser using the same EOMs to generate two sidebands spectrally separated from the control by the modulation frequency *ω*_*m*_. The probe laser frequency ${\omega }_{p}={\omega }_{c}+{\omega }_{m}$ can be swept using *ω*_*m*_ relative to the control laser *ω*_*c*_. An erbium-doped fiber amplifier (EDFA) is used after each EOM to independently modify the control laser power, which in turn regulates the pump-enhanced Brillouin coupling rate *G* in either direction. Fiber polarization controllers (FPC) are used to match the light polarizations of the forward and backward propagating laser fields. Two circulators are placed before and after the resonator to allow simultaneous measurements of the probe transmissions in the forward and backward directions without reconfiguring the experimental setup. We use a total of four photodetectors, two for measuring the forward and backward probes which are used as references (PD1 and PD2 in Fig. 6) and the other two for measuring the forward and backward probe transmissions through the resonator-waveguide system (PD3 and PD4 in Fig. 6). The experiment is performed at room temperature and atmospheric pressure condition.

## Electronic supplementary material

Supplementary information
