# Supplementary material for: Complete linear optical isolation at the microscale with ultralow loss
Source: Sci Rep. 2017 May 8;7:1647. doi: 10.1038/s41598-017-01494-w (PMC5431488; doi:10.1038/s41598-017-01494-w)
Supplement: Supplementary file 1 — Supplementary information [file 41598_2017_1494_MOESM1_ESM.pdf]

Supplementary information: Complete linear optical isolation at the microscale with ultralow loss

JunHwan Kim[†], Seunghwi Kim[†], Gaurav Bahl[*]

Mechanical Science and Engineering,

University of Illinois at Urbana-Champaign, Urbana, Illinois, USA

[†] Equal contribution.

[*] To whom correspondence should be addressed; E-mail: bahl@illinois.edu.

Classical description of induced transparency arising from light-sound coupling in a resonator

The coupled classical field equations for our waveguide-resonator system can be derived from the acoustic and electromagnetic wave equations under the slowly varying amplitude approximation, and a detailed explanation can be found in [S.1] and also the Supplement of Ref. [S.2].

$$\begin{aligned}
\dot{a_1} &= -(\kappa_1/2 + j\Delta_1)a_1 - j\beta^* u^* a_2 + \sqrt{\kappa_{\text{ex}}}\, s_{1,\text{in}} \\
\dot{a_2} &= -(\kappa_2/2 + j\Delta_2)a_2 - j\beta u a_1 + \sqrt{\kappa_{\text{ex}}}\, s_{2,\text{in}} \\
\dot{u} &= -(\Gamma_B/2 + j\Delta_B)u - j\beta^* a_1^* a_2 + \xi \\
s_{i,\text{out}} &= s_{i,\text{in}} - \sqrt{\kappa_{\text{ex}}}\, a_i \mid_{\text{where } i=1,2}
\end{aligned} \tag{S.1}$$

where a_i is the non-dimensional intracavity optical field at the control (i=1) or the probe (i=2) frequencies, u is the non-dimensional intracavity acoustic field, β is the acousto-optic coupling rate, κ_i is the loaded optical loss rate, Γ_B is the phonon loss rate, and κ_{ex} is the coupling rate between the waveguide and resonator. The loaded optical loss rates are defined as $\kappa_i = \kappa_{i,\text{o}} + \kappa_{ex}$ where $\kappa_{i,\text{o}}$ is the loss rate intrinsic to the optical mode. The Δ_i parameters are the field detuning (subscript B for the acoustic field), ξ is the thermal mechanical fluctuation (noise), and $s_{i,\text{in}}$ and $s_{i,\text{out}}$ are the optical driving and output fields in the waveguide respectively (Manuscript Fig. 1b). We can safely assume that $s_{1,\text{in}}$ is a stronger source for the control field a_1 compared against the scattering contribution. Further, the thermal fluctuation source ξ is negligible when we

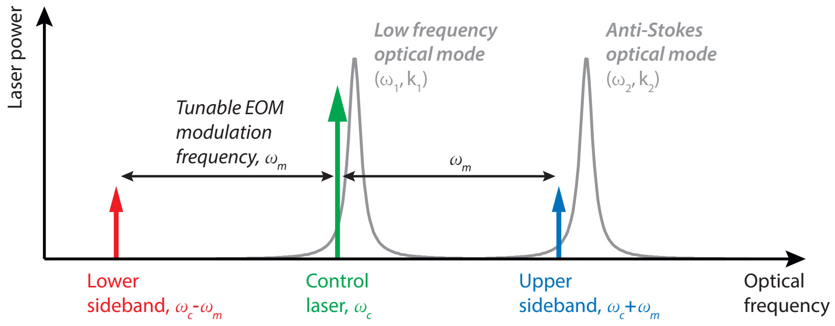

Supplementary Figure S.1: An electro-optic modulator (EOM) generates lower and upper sidebands at modulation offset ω_m away from the control laser. The upper sideband is used as a probe to measure transmission coefficient through the system. The lower sideband does not interact with any feature of interest in this system and transmits unhindered. The optical modes are thermally self-locked [S.4] to the control laser on the lower optical mode.

solve for time-averaged intracavity fields [S.3]. Solving Eqn. set S.1 for the probe output field $s_{2,\text{out}}$ given a probe driving field $s_{2,\text{in}}$ yields the steady-state probe transmission coefficient $\tilde{t}_p$.

$$\tilde{t}_p = \frac{s_{2,\text{out}}}{s_{2,\text{in}}} = 1 - \frac{\kappa_{\text{ex}}}{(\kappa_2/2 + j\Delta_2) + G^2/\left(\Gamma_B/2 + j\Delta_B\right)} \quad \text{(S.2)}$$

where $G = |\beta a_1|$ is the pump-enhanced Brillouin coupling rate. The coupling rate G is manipulated by the control driving field $s_{1,\text{in}}$ in the waveguide through the relation $a_1 = s_{1,\text{in}}\sqrt{\kappa_{\text{ex}}}/\left(\kappa_1/2 + j\Delta_1\right)$.

The shape of the function presented in Eqn. S.2 resembles the conventional optical absorption by a resonator, but with the acousto-optic interaction leading to a transparency within the absorption signature (see Fig. 2 in the Manuscript). As summarized in the Manuscript, the momentum dependence of the acousto-optic coupling rate β breaks the direction symmetry of this transparency. When the system is critically coupled and Brillouin acousto-optic coupling is engaged, the transmission $\tilde{t}_p$ can be shown to reach zero in one direction, with transmission in the opposite direction approaching 100%.

Determining the optical probe transmission coefficient

The probe transmission coefficient $\tilde{t}_p$ is measured with the help of a network analyzer, which performs a coherent ratiometric analysis of beat notes of the control and probe optical signals at various points in the experiment (see Fig. 3 in the Manuscript). Here, we dissect the network analyzer measurement to explain how the probe transmission coefficient is extracted from the experimental data.

The control laser with frequency ω_c is electro-optically modulated at ω_m to create two sidebands. By keeping the modulation depth low, we can ensure the sidebands are small compared to the carrier, allowing a first-order approximation of the spectrum. We can thus write the optical field within the fiber **prior to the resonator** as the following

$$\tilde{E}_{in} = E_c e^{-j\omega_c t}\left(1 + \frac{m}{2}e^{j\omega_m t} + \frac{m}{2}e^{-j\omega_m t}\right) + c.c. \tag{S.3}$$

where E_c is the carrier or control laser field amplitude and m is the modulation depth. The control laser frequency ω_c and modulation frequency ω_m are adjusted such that the control laser and upper sideband overlap with optical modes while the lower sideband does not couple to any of the resonator's optical modes (Fig. S.1). We use the upper sideband as the optical probe to measure light transmission through the system, while the control laser parked at the lower frequency optical mode enables the Brillouin scattering interaction between the optical mode pair.

The optical field arriving at the photodetector **after passing through the taper-resonator system** (PD3 in the forward case, PD4 in the backward case, see Fig. 3 in the Manuscript) can then be expressed as

$$\tilde{E}_{out} = E_c e^{-j\omega_c t}\left(\tilde{t}_c + \tilde{t}_{ls}\frac{m}{2}e^{j\omega_m t} + \tilde{t}_p\frac{m}{2}e^{-j\omega_m t}\right) + c.c. \tag{S.4}$$

where $\tilde{t}_i$ are the complex valued transmission coefficients of the control, lower sideband, and probe (upper sideband) fields (i=c, ls, p respectively) through the waveguide. Since the lower sideband does not couple to the resonator, its transmission coefficient is simply $\tilde{t}_{ls} = 1$ (Fig. S.1). The probe transmission coefficient $\tilde{t}_p$ measured in the forward and backward directions defines the optical isolation performance.

The optical power measured at the output detector (PD3 or PD4 depending on the probe direction) can be extracted from Eqn. S.4 as shown below. Here we consider only the terms that fall within the detector bandwidth at frequency ω_m.

$$\begin{aligned} P_{out} \propto \left|\tilde{E}_{out}\right|^2 &= |E_c|^2\, t_c \frac{m}{2}\left(e^{-j\omega_m t} + \tilde{t}_p e^{-j\omega_m t}\right) + c.c. \\ &= |E_c|^2\, t_c m\left[\ \left(1 + \mathrm{Re}(\tilde{t}_p)\right)\cos\omega_m t\ +\ \mathrm{Im}(\tilde{t}_p)\sin\omega_m t\ \right] \end{aligned} \tag{S.5}$$

Without loss of generality we have set $\tilde{t}_c$ with a phase of zero, i.e. all other fields are referenced to the phase of control field. The RF (electrical) output signal from the photodetector is P_{out} multiplied by the detector gain.

The network analyzer requires a reference signal to perform the ratiometric measurement. We generate this reference by directly measuring the optical signal prior to the resonator, i.e. Eq. S.3, at PD1 (PD2 in the backward direction, see Fig. 3 in the Manuscript). As above, this reference signal is proportional to the optical power

$$P_{ref}\ \propto\ 2\,|E_c|^2\, m\cos(\omega_m t) \tag{S.6}$$

With respect to this $\cos(\omega_m t)$ reference, the first term in the parentheses in Eq. S.5 provides the in-phase component $\left(1 + \mathrm{Re}(\tilde{t}_p)\right)$ of the measurement, while the second term provides the quadrature component $\mathrm{Im}(\tilde{t}_p)$. The network analyzer output typically converts this measurement to the polar form

$$Ae^{j\phi} = \frac{t_c M}{2}\left(1 + \tilde{t}_p\right) \tag{S.7}$$

where A is the amplitude response, ϕ is the phase response, and M is a coefficient accounting for a fractional difference between optical powers measured at the reference photodetector and at the photodetector placed after the resonator at a decoupled state. M includes EOM output power split ratio (1:99), EDFA gain, and difference in photodetectors' sensitivities.

The frequency and power of the control laser remain unchanged during the experiment, resulting in a constant t_c that can be determined by monitoring the control laser transmission. M and t_c can also be determined together through the network analyzer response when the probe is off resonance from the anti-Stokes mode, i.e. in the case where $\tilde{t}_p = 1$. Using this information, curve fitting can be performed on the network analyzer measurement, and the complex $\tilde{t}_p$ can be separately determined as a function of offset (from the control laser) frequency ω_m. However, we note that the transmission coefficient extracted using Eq. S.7 is not of the probe field only. We must also consider the effect of coherent light sources other than the probe for the accurate measurement of the true probe transmission coefficient. We discuss this in detail in the next section.

Background light in probe measurements

In ultra-high-Q resonators, coherent spontaneous light scattering from the control laser by small intrinsic defects can populate the anti-Stokes optical mode. There may also be direct injection of the control laser into the anti-Stokes optical mode. While this extra light is generally small, it does result in competition with the small amount of anti-Stokes light scattering from the acoustic mode in the structure (the phenomenon of interest) and can contaminate measurements of $\tilde{t}_p$. An exemplary measurement of this spurious light is shown in Fig. S.2. Since both light sources are being generated from the same pump/control laser, there can be interference that complicates the measurement of the probe transmission (Fig. S.3).

We can easily observe the evidence of this additional light in the background using an electrical spectrum analyzer that monitors the RF power measurement from the photodetector. Fig. S.2 shows the spectrum of beat notes generated from scattering by the acoustic phonon mode centered at $\Omega_m = 403.6$ MHz, and from background light within the anti-Stokes optical mode centered at $\omega_{as} = 405.1$ MHz offset from the control laser frequency. Since the resonant frequency of the phonon mode is fixed, the frequency of the beat note originating from Brillouin scattering does not change when the control laser frequency changes.

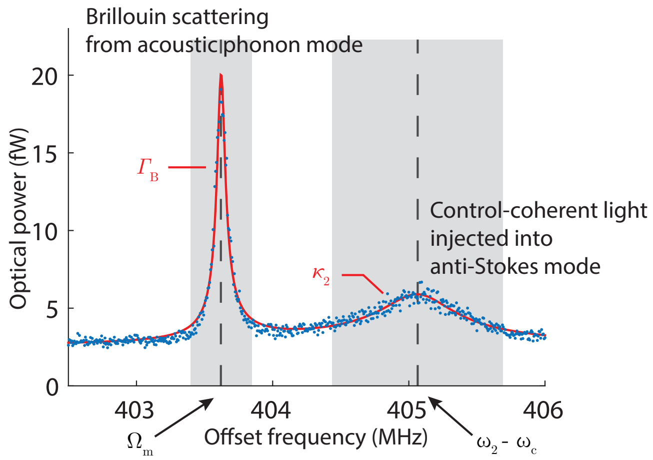

Supplementary Figure S.2: Measurement of Brillouin scattering at Ω_m and the control-coherent background light (from defect induced scattering and direct injection) shaped by the anti-Stokes optical mode at ω_2. Both sources are offset from the control laser by roughly 404 MHz. This measurement is derived from their beating with the control field on a photodetector and measured by an RF electronic spectrum analyzer. The additional background light is typically too small to be observed except when it is resonantly amplified by an ultra-high-Q resonator. The Brillouin scattering occurs at a fixed frequency defined by the phonon mode Ω_m while the background light is tunable by modifying the control laser frequency.

However, the beat note generated from the control-coherent background light injected into the anti-Stokes optical mode can be moved in frequency space with the control laser.

Since the proposed sources of background light (defect scattering, direct injection) are proportional to the intracavity control field a_1, we can model them as a coherent source driving the anti-Stokes mode with coupling strength r relative to the control field. This extra source can be included in the equations describing our system as follows :

$$\begin{aligned} \dot{a_1} &= -(\kappa_1/2 + j\Delta_1)a_1 - j\beta^* u^* a_2 + \sqrt{k_{ex}} s_{1,in} \\ \dot{a_2} &= -(\kappa_2/2 + j\Delta_2)a_2 - j\beta u a_1 + \sqrt{k_{ex}} s_{2,in} + jra_1 \\ \dot{u} &= -(\Gamma_B/2 + j\Delta_B)u - j\beta^* a_1^* a_2 + \xi \\ s_{i,out} &= s_{i,in} - \sqrt{k_{ex}}\, a_i \mid_{\text{where } i=1,2} \end{aligned} \tag{S.8}$$

As discussed in the Supplement §1, the control field scattering $j\beta^* u^* a_2$ and thermal fluctuation ξ can be assumed to be negligible. We find the modified steady-state intracavity probe field a_2 which is composed of the unperturbed

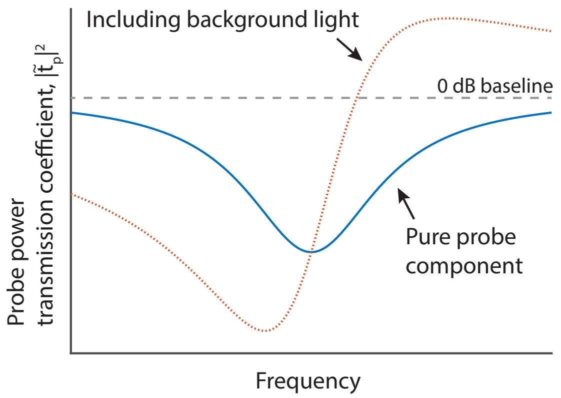

Supplementary Figure S.3: The network analyzer measurement shows an asymmetric probe power transmission coefficient $|\tilde{t}_p|^2$ (red-dotted line) in spite of the optical mode being symmetric. The distortion in the measurement originates from the background light injected to the anti-Stokes optical mode, creating difficulty in the estimation of the 0 dB transmission baseline (grey-dashed line) and optical mode center frequency. After correcting for the background light, the symmetric optical transmission is seen (blue-solid line).

probe response and an additional background light term:

$$\begin{aligned} a_2 &= \frac{\sqrt{k_{ex}} s_{2,in}}{\gamma_2 + G^2/\gamma_B} + \frac{jra_1}{\gamma_2 + G^2/\gamma_B} \quad \text{(S.9)} \\ \gamma_i &= \kappa_i/2 + j\Delta_i \\ \gamma_B &= \Gamma_B/2 + j\Delta_B \end{aligned}$$

where $G = |\beta a_1|$ is the pump-enhanced Brillouin coupling rate. The probe field arriving at the photodetector is then expressed as

$$\begin{aligned} s_{2,out} &= \left(1 - \frac{k_{ex}}{\gamma_2 + G^2/\gamma_B}\right) s_{2,in} - \left(\frac{jr\sqrt{k_{ex}}}{\gamma_2 + G^2/\gamma_B}\right) a_1 \quad \text{(S.10)} \\ &= \tilde{t}_{p,\text{actual}}\, s_{2,in} - \left(\frac{jr\sqrt{k_{ex}}}{\gamma_2 + G^2/\gamma_B}\right) a_1 \end{aligned}$$

implying that the measured probe transmission coefficient (by definition) will be

$$\begin{aligned} \tilde{t}_{p,\text{measured}} &= \frac{s_{2,out}}{s_{2,in}} \\ &= \tilde{t}_{p,\text{actual}} - \left(\frac{jr\sqrt{k_{ex}}}{\gamma_2 + G^2/\gamma_B}\right) \frac{a_1}{s_{2,in}} \quad \text{(S.11)} \end{aligned}$$

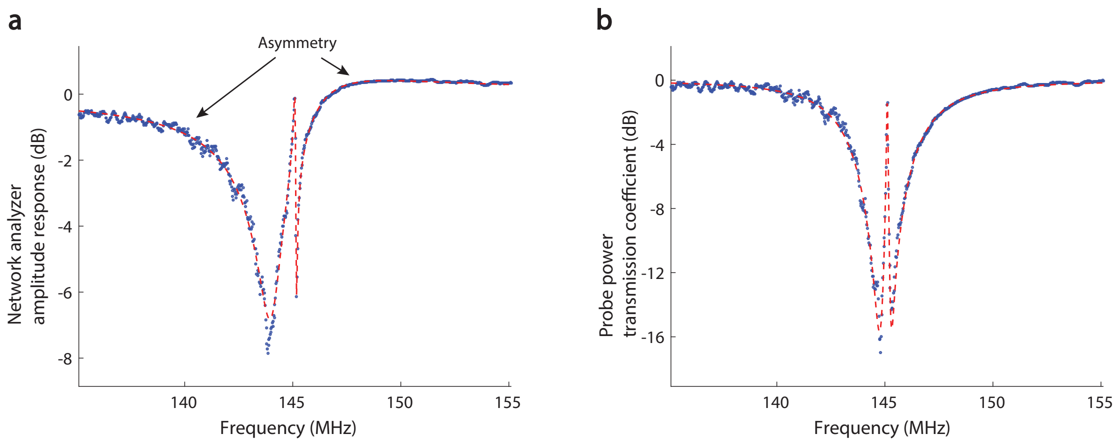

Supplementary Figure S.4: **a.** Raw amplitude response data from the network analyzer. The optical transmission measurement and the induced transparency are distorted by the additional light within the anti-Stokes optical mode. **b.** Probe optical power transmission is plotted using input-output relation after removal of the background light. Data shows transparency within the Lorentzian shaped optical mode. The red dashed line represents a fit using theoretical model for induced transparency.

From Eq. S.11, we note that we can reduce the effect of the background light by increasing the probe laser power (larger $s_{2,in}$) during the experiment such that the first term dominates. The measured transmission coefficient $\tilde{t}_{p,\text{measured}}$ acquired from Eq. S.7 will then approach the actual transmission coefficient $\tilde{t}_{p,\text{actual}}$. More generally, however, the second interfering term results in an asymmetry (irrespective of acousto-optical coupling) in the optical transmission through high-Q resonators measured by this pump-probe technique.

In Fig. S.3 we plot Eq. S.10 with acousto-optical coupling set to zero ($G = 0$). The extra light in the resonator modifies the transmission coefficient such that the high frequency side of the optical mode exceeds 0 dB baseline while the low frequency side is artificially dipped. When we exclude the background light, the plot now reveals a symmetric optical mode and a true resonance frequency. Thus, factoring out this asymmetry is critical in accurately determining the optical isolation performance. Unprocessed transmission measurement from the network analyzer (Fig. S.4a) shows the asymmetric optical mode shape and different 0 dB baseline levels on either side of the resonance. Such a mismatch in baseline is used to estimate the degree of asymmetry and the coupling strength r. The background light can then be subtracted from the measurement to obtain $\tilde{t}_{p,actual}$ as shown in Fig. S.4b. In the Fig. S.4 example, a symmetric optical mode with transparency at the center of the optical mode is revealed.

Comparison of isolation performance

We quantitatively compare isolation performance against previously demonstrated linear, magnet-free optical isolators in Supplementary Table S.1. Isolation contrast (extinction ratio), quantifies the ratiometric difference for the forward and reverse transmitted optical signals. Insertion loss quantifies the difference in the input and forward transmitted signals. All these approaches only operate over finite bandwidth, for which the 3 dB bandwidth quantifies the frequency span over which the contrast is within 3 dB of its highest value. We also provide device size and the system used to assist with determining the fit for specific applications. Since isolators may also be cascaded to increase contrast, we normalize the isolation contrast for each demonstration using 1 dB of insertion loss as a reference point, and provide the figure of merit as dB of contrast per 1 dB of insertion loss.

While many previous reports show signatures of optical nonreciprocity, several do not quantify the contrast or insertion loss metrics making it difficult to have a direct comparison. Sayrin et. al. report an excellent contrast per 1 dB of insertion loss from a resonantly enhanced spin-polarized cold atom system [S.5]. However, the target applications are different from the other magnet-free isolation approaches reported here as this requires laser cooling of the system. Lira et. al. report an impressive bandwidth of 200 GHz, but only show 3 dB contrast and extremely high insertion loss [S.6].

Our result exhibits an enormous 78.6 dB contrast per 1 dB of insertion loss, which rivals the values seen on commercial magneto-optic based optical isolators. Our demonstration has 69.5 dB higher figure of merit (nearly 7 orders-of-magnitude) relative to the next best microscale isolator result [S.5].

Supplementary Table S.1: Comparison of isolation performance for experimentally demonstrated non-magnetic linear non-reciprocal systems.

Author	Technique	Isolation contrast	Insertion loss	Contrast (dB) per 1 dB insertion loss (dB/dB)	3 dB Bandwidth	Device size	System used (laser wavelength)
Isolation using photonic microdevice							
This work	BSIT	11 dB	0.14 dB	78.6	400 kHz	170 µm	Microsphere (1550 nm)
H. Lira et. al. [S.6]	Interband scattering	3 dB	70 dB	0.0429	200 GHz	110 µm	Waveguide on-chip (1550 nm)
J. Kim et. al. [S.2]	BSIT	N/A	N/A	N/A	16.9 kHz	150 µm	Microsphere (1550 nm)
C.-H. Dong et. al. [S.7]	BSIT	N/A	N/A	N/A	9 kHz	196 µm	Microsphere (1550 nm)
Z. Shen et. al.[S.8]	OMIT	N/A	N/A	N/A	22 kHz	36 µm	Microsphere (780 nm)
B. Peng et. al. [S.9]	PT symmetry breaking	N/A	N/A	N/A	N/A	60 µm †	Toroid on-chip (1550 nm)
L. D. Tzuang et. al. [S.10]	Photonic Aharonov-Bohm effect	2.4 dB	N/A	N/A	N/A	325 µm	Waveguide on-chip (1550 nm)
Isolation through atom interactions							
C. Sayrin et. al. [S.5]	Scattering from spin-polarized cold atoms	7.8 dB	1.08 dB	7.22	N/A	0*	Atoms on tapered fiber (852 nm)
C. Sayrin et. al. [S.5]	Same as above with resonant enhancement	13 dB	1.43 dB	9.09	N/A	36 µm	Atoms on resonator (852 nm)
Isolation in macroscale fiber system							
M. S. Kang et. al. [S.11]	Stimulated Brillouin scattering	20 dB	N/A	N/A	7 MHz	15 m	Photonic crystal fiber (1550 nm)

† Requires two resonators of size 60 um.
* Size of an atom.
N/A = Not available.

References

[S.1] G. S. Agarwal and S. S. Jha, “Multimode phonon cooling via three-wave parametric interactions with optical fields,” *Phys. Rev. A*, vol. 88, p. 013815, Jul 2013.

[S.2] J. Kim, M. C. Kuzyk, K. Han, H. Wang, and G. Bahl, “Non-reciprocal Brillouin scattering induced transparency,” *Nature Phys.*, vol. 11, no. 3, pp. 275–280, Mar. 2015.

[S.3] S. Weis, R. Rivière, S. Deléglise, E. Gavartin, O. Arcizet, A. Schliesser, and T. J. Kippenberg, “Optomechanically induced transparency.” *Science*, vol. 330, no. 6010, pp. 1520–1523, Dec. 2010.

[S.4] T. Carmon, L. Yang, and K. J. Vahala, “Dynamical thermal behavior and thermal self-stability of microcavities,” *Opt. Express*, vol. 12, no. 20, pp. 4742–4750, Oct 2004.

[S.5] C. Sayrin, C. Junge, R. Mitsch, B. Albrecht, D. O’Shea, P. Schneeweiss, J. Volz, and A. Rauschenbeutel, “Nanophotonic Optical Isolator Controlled by the Internal State of Cold Atoms,” *Phys. Rev. X*, vol. 5, no. 4, p. 041036, Dec. 2015.

[S.6] H. Lira, Z. Yu, S. Fan, and M. Lipson, “Electrically Driven Nonreciprocity Induced by Interband Photonic Transition on a Silicon Chip,” *Phys. Rev. Lett.*, vol. 109, no. 3, p. 033901, Jul. 2012.

[S.7] C.-H. Dong, Z. Shen, C.-L. Zou, Y.-L. Zhang, W. Fu, and G.-C. Guo, “Brillouin-scattering-induced transparency and non-reciprocal light storage,” *Nature Commun.*, vol. 6, no. 6193, Feb. 2015.

[S.8] Z. Shen, Y.-L. Zhang, Y. Chen, C.-L. Zou, Y.-F. Xiao, X.-B. Zou, F.-W. Sun, G.-C. Guo, and C.-H. Dong, “Experimental realization of optomechanically induced non-reciprocity,” *Nature Photon.*, vol. 10, pp. 657–661, Aug. 2016.

[S.9] B. Peng, Ş. K. Özdemir, W. Chen, F. Nori, and L. Yang, “What is and what is not electromagnetically induced transparency in whispering-gallery microcavities,” *Nature Commun.*, vol. 5, no. 5082, Oct. 2014.

[S.10] L. D. Tzuang, K. Fang, P. Nussenzveig, S. Fan, and M. Lipson, “Non-reciprocal phase shift induced by an effective magnetic flux for light,” *Nature Photon.*, vol. 8, no. 9, pp. 701–705, Sep. 2014.

[S.11] M. S. Kang, A. Butsch, and P. S. J. Russell, “Reconfigurable light-driven opto-acoustic isolators in photonic crystal fibre,” *Nature Photon.*, vol. 5, no. 9, pp. 549–553, Sep. 2011.
